# Potassium Bromide in Orchitis

**Published:** 1882-04

**Authors:** 


### POTASSIUM BROMIDE IN ORCHITIS AND INFLAMED BREASTS.

J. Grammer, M.D., says that, when consulted in time, he finds nothing else necessary, either in orchitis or milk-breast, but potassium bromide in five-grain doses three times a day, or smaller doses more frequently repeated. In advanced or complicated cases a course of auxiliary measures should be used if only as a precaution or to expedite the cure ; but he has never had the bromide to fail him even when used alone. In orchitis a suspensory should always be worn. In some of these cases he has seen the disease held in abeyance for weeks, when the patients would persist in the grossest imprudence in walking and horseback-riding. He rarely restricts them in diet. Yet even these cases eventually recovered, without suppuration or atrophy, neither of which results has he seen since he has used this remedy. He has had no opportunity to test it in the metastatic orchitis or mumps, but is sure it will prove as useful here as in the ordinary cases ; and though the inflammation is specific, he expects to find the remedy efficient in the next epidemic of parotiditis he may meet with.

Dr. Grammer has seen but one case of mammary abscess since he commenced the use of the bromide of potassium for such cases, and that case occurred not long ago. The abscess had already pointed when he first saw it. He opened it and prescribed potassium bromide (two grains) every three hours during the day, and in less than a week her husband reported the patient well. This, however, was not a fair test of the effect of the bromide on a mammary abscess, for there was no infant to complicate or irritate the inflammation. It was to Dr. Grammer a unique instance of the secretion of milk during pregnancy. The woman was four or five months advanced with her fourth child, and she stated that, being habitually rather irregular, she always recognized her pregnancy by the appearance of milk, the secretion of which thenceforth continued.—*Virginia Med. Monthly.*

